# Accuracy of the pressure-volume curve method compared to quantitative lung CT scan to assess the recruitable lung in patients with acute respiratory failure

**DOI:** 10.1186/cc10711

**Published:** 2012-03-20

**Authors:** D Chiumello, A Marino, I Cigada, F Menga, M Brioni, IR Piva

**Affiliations:** 1 Fondazione IRCCS Ca' Granda - Ospedale Maggiore Policlinico, Milan, Italy; 2 Università degli Studi di Milano, Milan, Italy

## Introduction

In patients with acute lung injury the knowledge of recruitable lung is useful for a physiological PEEP setting. The quantitative lung CT scan analysis remains the reference method [1]. However, it is time consuming and often it is not applicable in clinical management. The PV curve at two PEEP levels has been proposed as an alternative method [2]. The aim of this study was to evaluate the accuracy of these two methods in predicting the lung recruitability.

## Methods

Sedated and paralyzed patients underwent a PV curve using the low-flow method and whole-lung CT scan at 5 and 15 cmH_2_O of PEEP. The lung recruitability was defined as the decrease in the not aerated tissue by the quantitative lung CT analysis and as the difference between the lung volume computed on the two PV curves for an airway pressure of 20 cmH_2_O.

## Results

**Figure 1:**
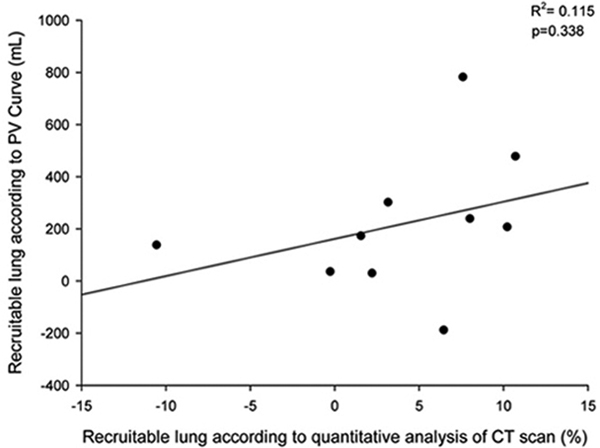
**Linear regression between quantitative CT scan analysis and PV curve method**.

Ten patients (mean age 65.4 ± 10.4 years, body mass index 24.0 ± 6.8 kg/m^2^, PaO_2_/FiO_2 _181 ± 37) were enrolled. The mean recruitable lung was 3.9 ± 6.3% of the total lung weight and 218 ± 266 ml for the quantitative lung CT scan and PV curve. The linear regression between the two methods (Figure 1) was not significant (*P *= 0.338 and *R*^2 ^= 0.115).

## Conclusion

The recruitable lung computed as the difference in not aerated tissue was not related to the difference in volume estimated by the PV curve. The role of the PV curve to estimate the lung recruitability remains to be elucidated.
